# Concurrent treatment with simvastatin and *NF-κB* inhibitor in human castration-resistant prostate cancer cells exerts synergistic anti-cancer effects via control of the *NF-κB/LIN28/let-7 miRNA* signaling pathway

**DOI:** 10.1371/journal.pone.0184644

**Published:** 2017-09-14

**Authors:** Minyong Kang, Kyoung-Hwa Lee, Hye Sun Lee, Chang Wook Jeong, Ja Hyeon Ku, Hyeon Hoe Kim, Cheol Kwak

**Affiliations:** 1 Department of Urology, Samsung Medical Center, Sungkyunkwan University School of Medicine, Seoul, Republic of Korea; 2 Department of Urology, Seoul National University Hospital, Seoul, Republic of Korea; Roswell Park Cancer Institute, UNITED STATES

## Abstract

We examined the anti-cancer effects and molecular mechanism of simvastatin in human castration-resistant prostate cancer (CRPC) cells, particularly focused on *LIN28B* and its target molecule, *let-7* microRNA (miRNA) among the various target genes of NF-κB. A human CRPC cell line (PC3) was used in the current study. Gene expression patterns were evaluated using real time-PCR and western blot analysis. CCK-8 assay was used for assessing cell viability and proliferation, and a clonogenic assay was adopted to evaluate clonal proliferative capabilities. Induction of apoptotic cell death was analyzed via flow cytometry. Small interfering RNA (siRNA) and short-hairpin RNA (shRNA) were used for manipulating the expression of genes of interest. PC3 showed relatively higher expression levels of *LIN28B* and lower expression levels of *let-7* miRNAs. Simvastatin treatment significantly inhibited cell viability and clonal proliferation in a dose-dependent manner. Importantly, the downregulated *let-7* miRNA family was restored after simvastatin treatment. We further observed that human CRPC cells transfected with *LIN28B*-siRNA or shRNA also showed upregulated *let-7* miRNAs. Finally, dual treatment with simvastatin and an *NF-κB* inhibitor (CAPE) synergistically induced apoptotic cell death, along with reduction of *LIN28B* expression, and restoration of *let-7* miRN*As* levels. Our data illustrate that simvastatin remarkably inhibits the growth of human CRPC cells by suppressing *NF-κB* and *LIN28B* and subsequently upregulating *let-7* miRNAs. Moreover, concurrent treatment with simvastatin and an NF-κB inhibitor synergistically suppressed the growth of human CRPC cells, suggesting a novel therapeutic approach for human CRPC treatment.

## Introduction

The incidence of prostate cancer (PCa) has increased rapidly over the decades and has become a crucial health issue world-wide [1]. PCa gradually progresses over time and shows a low cancer-specific mortality [2]. However, if patients with PCa progress to castration-resistant prostate cancer (CRPC), they mostly die within 24 months after the diagnosis of CRPC [3]. Although systemic chemotherapy and/or androgen receptor (AR)-targeted agents are regarded as treatments of choice for CRPC, treatment is hindered by adverse effects and drug-resistance [4]. In this context, development of alternate agents with good efficacy and minimal adverse effects is urgently needed for treating patients with CRPC.

One of the promising approaches is targeting the aberrant metabolism of cancer cells without damaging normal cells by using specific agents that control metabolic disorders, such as statins [5]. Statins primarily inhibit 3-hydroxy-3-methylglutaryl-coenzyme A (HMG-CoA) reductase within the intracellular cholesterol biosynthesis pathway, and are widely used for treating hypercholesterolemia [6]. In addition to the accumulating evidence for the anti-cancer efficacy of statins, we have found that human CRPC cells (PC3 and DU145) show high expression of NF-κB and that simvastatin treatment induces apoptotic cell death by downregulation of activated NF-κB signaling [7]. However, the detailed molecular mechanisms underlying the anti-cancer effects of simvastatin remain unclear.

Among various downstream genes of the *NF-κB* signaling pathway, *LIN28B* has received great interest as a key oncogene, because it specifically blocks the biogenesis of *let7*-miRNA, which inhibits a number of oncogenic target genes such as Myc, Ras, and cyclins [8]. In this study, we focused on *LIN28B* and its target molecule, *let7*-microRNA (miRNA) as the key molecular mechanism underlying the anti-cancer effects of statins in human CRPC. In this study, we hypothesized that the deregulated *NFκB-Lin28B-let7-miRNA* signaling pathway can be restored by statin treatment and suppress the growth and proliferation of human CRPC cells.

## Materials and methods

### Cell culture and reagents

PC3, a well-known human CRPC cell line, was used in the current study. PC3 was purchased from the American Type Culture Collection (Rockville, MD, USA). This cell line was cultured in RPMI-1640 medium (WELGENE, Gyeongsan, Korea) supplemented with 10% fetal bovine serum (FBS; BIOWEST, Nuaillé, France), 1% penicillin-streptomycin (Thermo Fisher Scientific, MA USA), and 1% nonessential amino acids (Invitrogen) at 37°C with 5% CO_2_. The details of the primers and primary antibodies used in our study are presented in Tables 1 and 2, respectively.

**Table 1:** Details of the primary antibodies used in the present study.

Antibody	Host	Dilution factor	Industry
Lin28B	Rabbit	1:1000	Cell Signaling
Lin28B	Mouse	1:1000	Millipore
β-Actin	Mouse	1:10000	Sigma-Aldrich
NF-κB	Rabbit	1:2000	Cell Signaling
Cyclin D1	Rabbit	1:1000	Cell Signaling
c-PARP	Rabbit	1:1000	Cell Signaling

**Table 2:** Details of real time RT-PCR primers.

Gene	Forward primer sequence (5′-3′)	Reverse primer sequence (5′-3′)
*Let7a_QuantiM*	tga ggt agt agg ttg tat agt t	-
*Let7b_QuantiM*	tga ggt agt agg ttg tgt ggt t	-
*Let7c_QuantiM*	tga ggt gat agg ttg tgt ggt t	-
*Let7d_QuantiM*	aga ggt agt agg ttg cat agt t	-
*Let7e_QuantiM*	tga ggt agg agg ttg tat agt t	-
*Let7f_QuantiM*	tga ggt agt aga ttg tat agt t	-
*Let7g_QuantiM*	tga ggt agt agt ttg tac agt t	-
*hmiR-98*	tga ggt agt aag ttg tat tgt t	-
*U6*	tga ggt agt aag ttg tat tgt t	-
*Lin28B*	gca aag gtg gtg gag aag ag	ggc ttc cct ctc ggt tta tc
*CyclinD1*	gag tga tca agt gtg acc cgg a	tgg ggt cca tgt tct gct gg
*GAPDH*	gag aag gct ggg gct cat	tgc tga tga tct tga ggc tg
*18S ribosomal RNA*	ttc gta ttg agc cgc tag a	ctt tcg ctc tgg tcc gtc tt

### RNA isolation and real-time PCR (q-PCR)

After isolating total RNA using the TRIsure (BIOLINE, London, UK) solution and the SV Total RNA Isolation System (Promega, Wisconsin, USA), complementary DNA (cDNA) was synthesized using TOPscript^™^ DryMIX(dN6 plus) from Enzynomics (Daejeon, Korea). For the real-time polymerase chain reaction (q-PCR), the EvaGreen q-PCR Master Mix Kit (Applied Biological Materials Inc., Richmond, BC, Canada) was used with the StepOneTM Real-time PCR System (Applied Biosystems). Relative transcriptional expression of the target genes was calculated by the 2^-ΔΔCt^ method using 18S ribosomal RNA as the internal control. Moreover, the reverse transcriptions of the *let7*-miRNA family were performed using the miRNA cDNA synthesis kit (Applied Biological Materials Inc) according to the manufacturer’s instructions. All the primers for qPCR were designed and purchased from Bioneer (Daejeon, Korea).

### Western blotting

Treated CRPC cells were lysed in EzRIPA lysis buffer (ATTO, Taito-ku, Tokyo) with the protease inhibitors/ phosphatase inhibitors cocktail to prepare the protein samples. Protein concentration was calculated using a Pierce^®^ BCA Protein Assay kit (Thermo Fisher Scientific). Whole cell lysates in sample buffer were loaded and run on sodium dodecyl sulfate (SDS)-polyacrylamide gels, and transferred to an Immobilon-P membrane (Millipore, Darmstadt, Germany). For blocking, the membranes were incubated with 4% skim milk solution in TBS-T (0.05% Tween-20) for 1 h. Primary antibodies were incubated with the membranes overnight at 4°C, followed by incubation with a horseradish peroxidase–conjugated secondary antibody (1:5,000) for 30 min. Membranes were finally developed using the ECL-Plus Kit (Thermo Fisher Scientific), and the results were obtained under a LAS 4000 biomolecular imaging system (GE healthcare life science).

### LIN28B-siRNA transfection and generation of LIN28B knockdown cells

*LIN28B-*shRNA (no. 1, no. 2; Sigma-Aldrich, MO, USA) and scramble-shRNA plasmids (no 1; Sigma-Aldrich) were transfected into PC3 cells. To establish stable *LIN28B*-knockdown cells, the transfected cells were cultured in medium containing selective antibiotics for 14 to 21 d. After selection, the knockdown efficiency was examined by q-PCR and western blot analysis. Scramble-shRNA transfected cells were used as the negative control for *LIN28B*- knockdown cells.

### Cell viability and cell proliferation analyses

Cells (2,000 to 3,000 cells/well) were dispensed in 100 μL culture medium in a 96-well plate, and incubated for 24 h in a humidified incubator at 37°C with 5% CO_2_. To perform cell viability analysis, EZ-CYTOX (DOGEN, Seoul, Korea) was used. After 24 to 72 h treatment with various concentrations of simvastatin (0, 5, 10, 20, and 40 μL), EZ-CYTOX solution (10 μL) was mixed with the culture medium in each well. Samples were incubated for 1 h at 37°C. We measured the absorbance of each sample at 450 nm using a microplate reader (PerkinElmer, Waltham, MA). Cell viability data were presented as percentage values for each treatment condition compared to that of the control.

For the cell proliferation assay, we measured cell viabilities at the 0, 24, 48, and 72 h time points, respectively, according to the each experimental condition during three days of culture. We used non-treated cells or scramble-shRNA transfected cells as negative controls. All the data on growth curves are indicated as fold change compared to the results from the initial day of culturing.

### Clonogenic assay

To investigate the abilities of clonogenic proliferation, human CRPC cells (1.5–2.0×10^3^/well) were plated onto 6-well plates and cultured for 10 to 14 days to observe significant colony formation, which comprises more than 50 individual CRPC cells. Visible colonies were stained using 0.01% crystal violet solution following fixation with 10% neutral buffered formalin solution (Sigma-Aldrich). After the samples were washed thrice with PBS, all the colonies were manually counted using a SZX7 stereo microscope (Olympus, Tokyo, Japan).

### Apoptosis analysis using flow cytometry

To perform the apoptosis assay, we used propidium iodide (PI) and an Annexin V-FITC detection kit (BD Biosciences, CA, USA). Treated and untreated cells under various conditions were prepared and resuspended at a concentration of 1 × 10^6^ cells/mL in the binding buffer. Samples were stained with PI and Annexin V-FITC for 30 min at room temperature in the dark. The proportion of apoptotic cell populations from each sample was detected using the BD FACSCalibur cytometer (BD Biosciences).

### Statistical analysis

All experiments were repeated thrice independently with technical replicates. The significant differences between experimental groups were statistically determined using the Student’s *t*- test or one-way ANOVA and multiple comparisons with post hoc tests. Results are presented as mean ± standard error of the mean (S.E.M). When the *p-*value was less than 0.05, we rejected the null hypothesis of no difference between experimental groups. SPSS Statistical program version 22.0 (SPSS, Inc., Chicago, IL, USA) and GraphPad Prism software ver. 5 (GraphPad Software Inc., San Diego, CA) were utilized for all statistical analyses in the present study.

## Results

### Human CRPC cells show higher LIN28B and lower let-7 miRNA family expression

**Fig 1:**
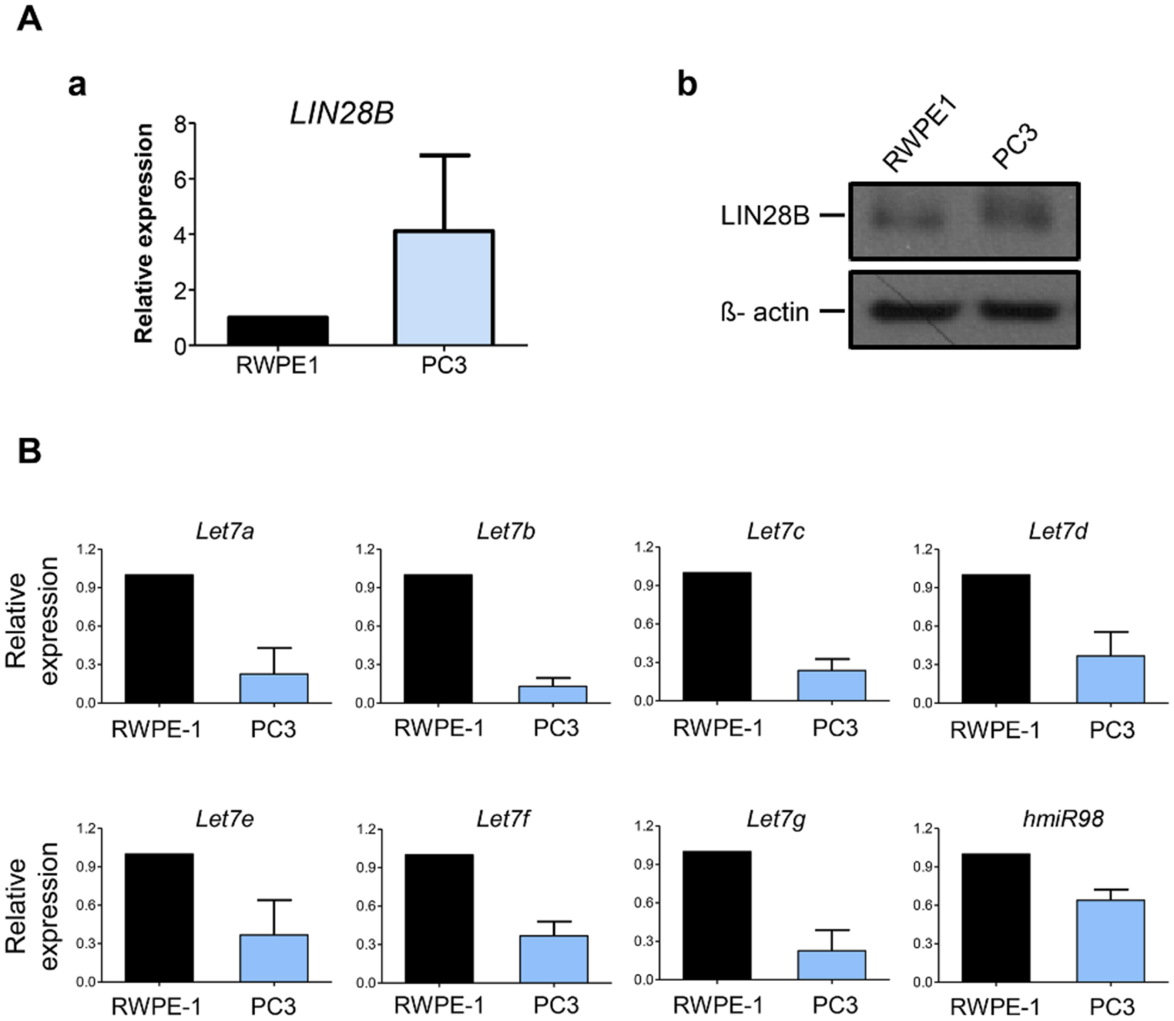
Expression patterns of *LIN28B* and the *let7*-microRNA (miRNA) family in human castration-resistant prostate cancer (CRPC) cells. **A.** Comparison of *LIN28B* expression levels in human CRPC cells (PC3) compared to those in the normal prostate cell line (RWPE1) by (a) quantitative reverse transcriptase-polymerase chain reaction (qPCR) for *LIN28B* mRNA expression and (b) western blot analysis for protein expression. In qPCR, relative mRNA expression is normalized to *GAPDH* expression levels, and finally presented in fold change as mean ± S.E.M (*n* = 3). In western blot analysis to determine the relative expression of LIN28B protein, beta-actin was used as the loading control. **B.** Comparison of let7-miRNA expression between RWPE1 and PC3 cell lines by qPCR analysis. Relative *miRNA* expression is normalized to *U6* expression levels, and finally presented in fold change as the mean ± S.E.M (*n* = 3).

We found that PC3, a well-known human CRPC cell line, showed relatively higher mRNA and protein expression of *LIN28B* compared to those in normal prostate cells (RWPE-1), respectively (Fig 1A-a and 1A-b). Similarly, other human CRPC cell lines, 22Rv1 and C4-2B, also showed significantly higher expression of *LIN28B* in mRNA and protein levels (S1 Fig). Conversely, we confirmed that human CRPC cells had significantly lower expression levels of all *let-7 miRNA* family members compared to those in RWPE-1 cells using qPCR analysis (Fig 1B). These results indicate that human CRPC cells had significantly upregulated *LIN28B* and subsequently downregulated *let-7 miRNA* families compared to those in normal control cells at the basal levels.

### Statin treatment reduced cell proliferation via inhibition of LIN28B and restoration of let-7 miRNA expression in human CRPC cells

**Fig 2:**
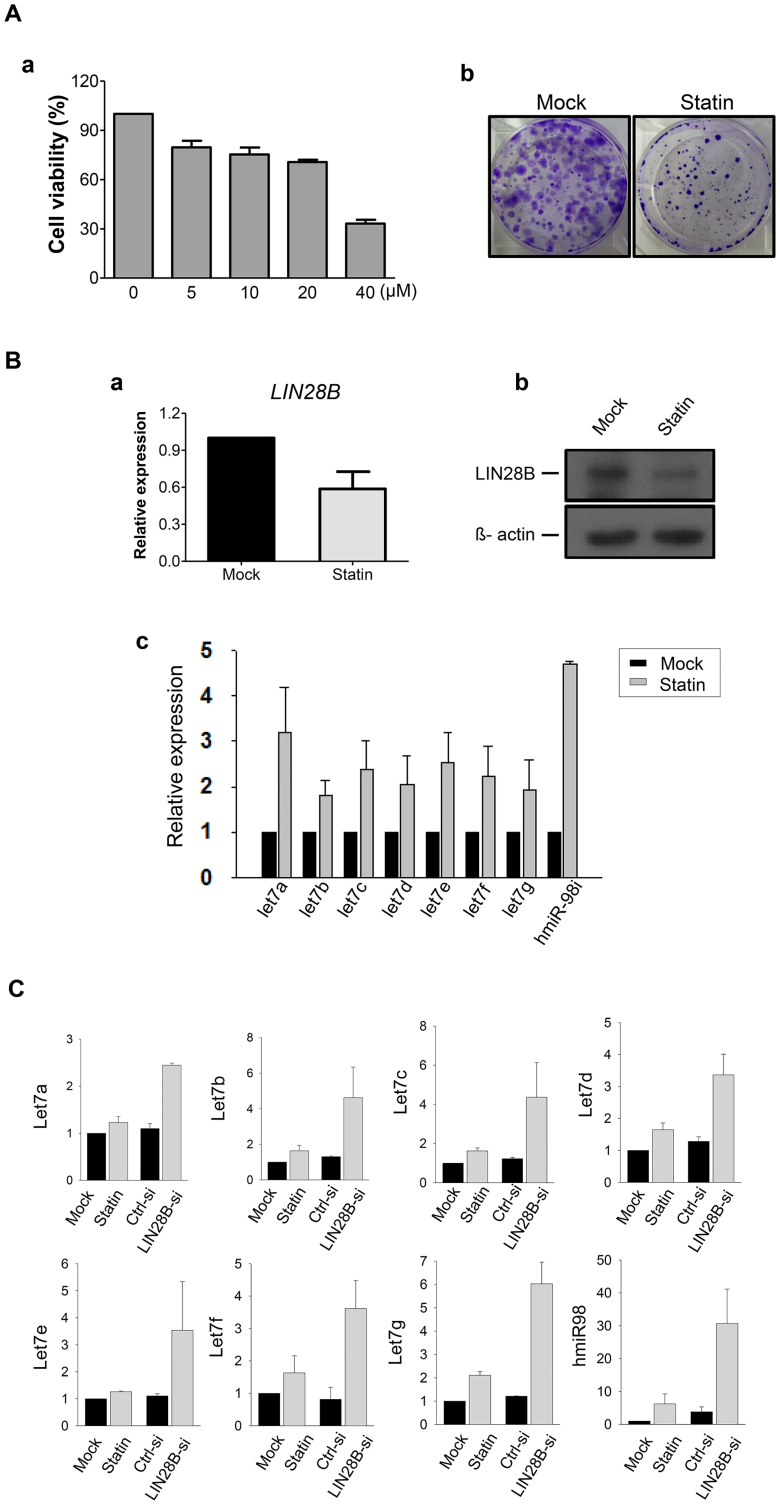
Anti-cancer effects of simvastatin by suppression of LIN28B and upregulation of let7 microRNAs in human CRPC cells. **A**. (a) Cell viability analysis according to different dosages of simvastatin (0, 5, 10, 20, and 40 μM) at 24 h in human CRPC cells (PC3). Values are shown as mean percentage of control ± S.E.M (*n* = 3). (b) Clonogenic assay according to simvastatin treatment (20 μM) in PC3 cells. Crystal violet staining was performed for showing colonies consisting of more than 50 individual cells. **B**. Comparison of *LIN28B* expression levels between control (mock) and simvastatin (20 μM for 24 h) treated PC3 cells by (a) quantitative reverse transcriptase-polymerase chain reaction (qPCR) and (b) western blot analysis. Relative transcriptional expression was normalized to *GAPDH* expression levels, and shown in fold change as mean ± S.E.M (*n* = 3). Beta-actin was used as the loading control for determining protein expression. (c) Comparison of *let7-miRNA* expression by qPCR between control (mock) and simvastatin (20 μM for 24 h) treated PC3 cells. Relative *miRNA* expression was normalized to *U6* expression levels, and shown in fold change as mean ± S.E.M (*n* = 3). C. Comparison of *let7-miRNA* expression by qPCR between the control (mock) and simvastatin (20 μM) treated PC3 cells, as well as scramble siRNA (Ctrl-si) and LIN28B-siRNA (LIN28B-si) transfected PC3 cells. Relative *miRNA* expression is normalized to *U6* expression levels, and illustrated by fold changes as mean ± S.E.M (*n* = 3).

After simvastatin treatments at various concentrations (0, 5, 10, 20, and 40μM) for 24 h in human CRPC cells, we observed that cell viabilities were significantly decreased in a dose-dependent manner (Fig 2A-a). Moreover, simvastatin treatment markedly suppressed clonal proliferation compared to those without treatments as shown in the clonogenic assay (Fig 2A-b). We also confirmed that simvastatin significantly reduced cell viabilities and clonogenic proliferation in other human CRPC cells, 22Rv1 and C4-2B, in a dose dependent manner (S2 Fig).

To address the molecular mechanism underlying the anti-cancer effects of simvastatin on human CRPC cells, we examined the changes in LIN28B and let-7 miRNA expression patterns upon simvastatin treatment. Notably, we observed that *LIN28B* expression was specifically suppressed by simvastatin administration in qPCR and western blot analysis (Fig 2B-a and 2B-b, respectively). More importantly, the downregulated *let-7* miRNA family expression in human CRPC cells increased after treatment with simvastatin (Fig 2B-c). We also confirmed that human CRPC cells transfected with *LIN28B*-siRNA also showed upregulated *let-7* miRNAs compared to those transfected with scramble-siRNA (Fig 2C). Furthermore, we found that LIN28B over-expression restored the simvastatin-induced cell death, showing higher cell viability compared to the control cells following 10 uM of simvastatin treatment (S3 Fig). Our data exhibited that simvastatin remarkably inhibited the growth of human CRPC cells by suppressing *LIN28B* and subsequently increasing *let-7* miRNAs.

### NF-κB inhibitor synergistically increased the anti-cancer effects of statin on human CRPC cells

**Fig 3:**
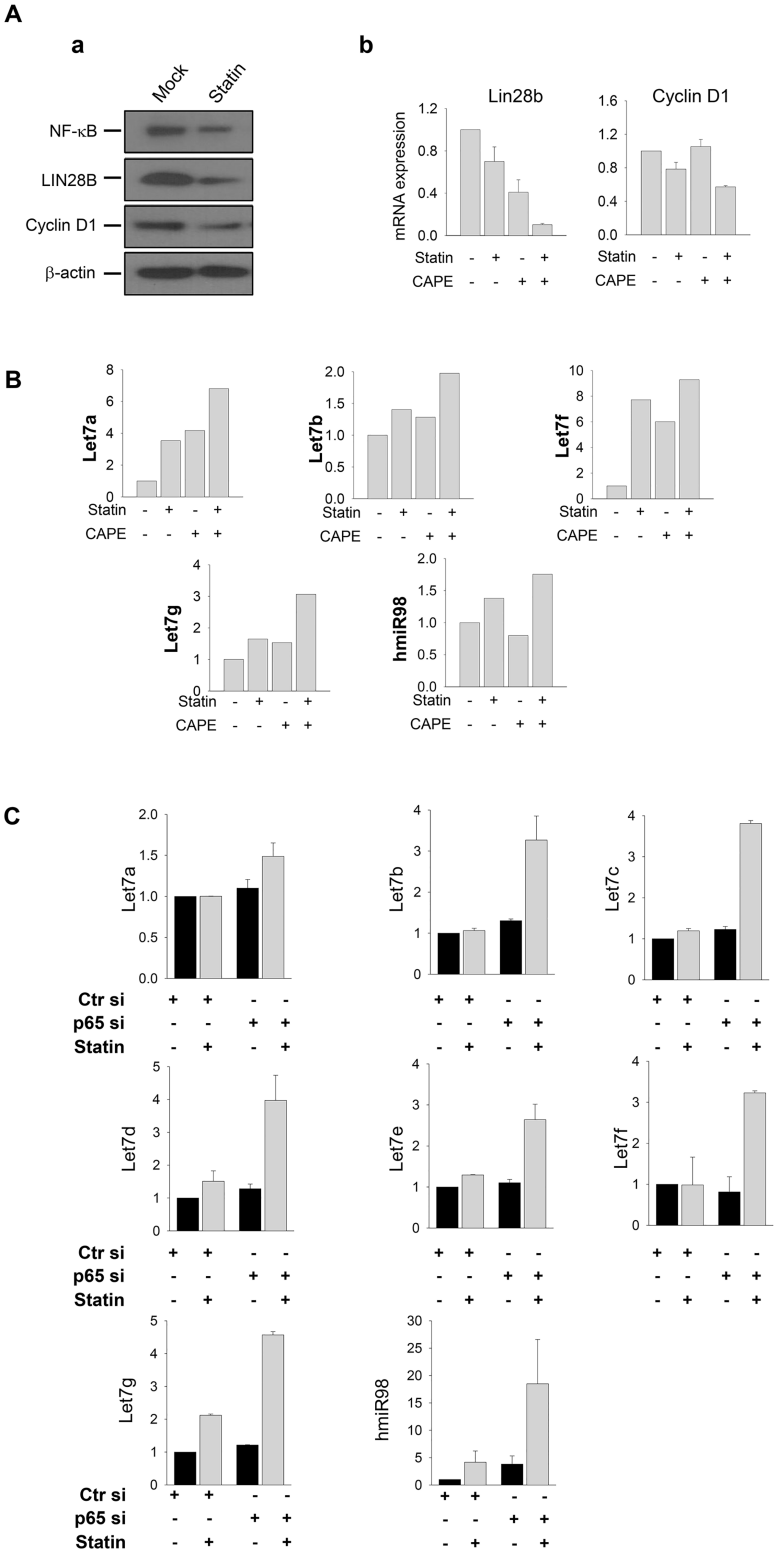
Synergistic suppression of LIN28B and restoration of let7-miRNA by concurrent treatment with simvastatin and an NF-κB inhibitor in human CRPC cells. **A**. (a) Comparison of expression patterns of *NF-κB-LIN28B-Cyclin D1* signaling axis according to the simvastatin treatment (20 μM for 24 h) by western blot analysis. Beta-actin was used as the loading control. (b) Effects on the transcriptional expression of LIN28B and Cyclin D1 as *NF-κB* target genes according to treatments of simvastatin and the *NF-κB* inhibitor, CAPE, in qPCR analysis. The combination of treatments is presented as “+” or “-” below the x-axis. Relative mRNA expression was normalized to *GAPDH* expression levels, and represented in fold change as the mean ± S.E.M (*n* = 3). **B.** Comparison of *let7-miRNA* expression according to the treatments of simvastatin and the *NF-κB* inhibitor CAPE by qPCR analysis. The combination of treatments is presented as “+” or “-” below the x-axis. Relative *miRNA* expression is normalized to *U6* expression levels, and presented by fold changes as the mean ± S.E.M (*n* = 3). **C.** Comparison of *let7-miRNA* expression according to the treatments of simvastatin and transfection of *p65-siRNA* for NF-κB inhibition by qPCR analysis. Scramble siRNA (Ctr si) was used as a negative control for p65-siRNA transfection. Combination of treatments is presented as “+” or “-” below the x-axis. Relative *miRNA* expression is normalized to *U6* expression levels, and shown by fold change as the mean ± S.E.M (*n* = 3).

We next explored the association between *NF-κB* and the *LIN28-let7-*miRNA signaling axis as the molecular mechanism underlying the anti-cancer effects of simvastatin on human CRPC cells. As shown in Fig 3A-a, simvastatin treatment reduced the expression of *NF-κB* as well as *LIN28B* in western blot analysis. The expression of *cyclin D1*, as a downstream target gene of this signaling pathway was also attenuated by simvastatin administration (Fig 3A-a). Importantly, we found that the degree of *LIN28B* and *cyclin D1* downregulation was synergistically reduced by the combined treatment with simvastatin and the *NF-κB* inhibitor (CAPE) compared to that with single treatments of either simvastatin or CAPE alone in human CRPC cells (Fig 3A-b).

**Fig 4:**
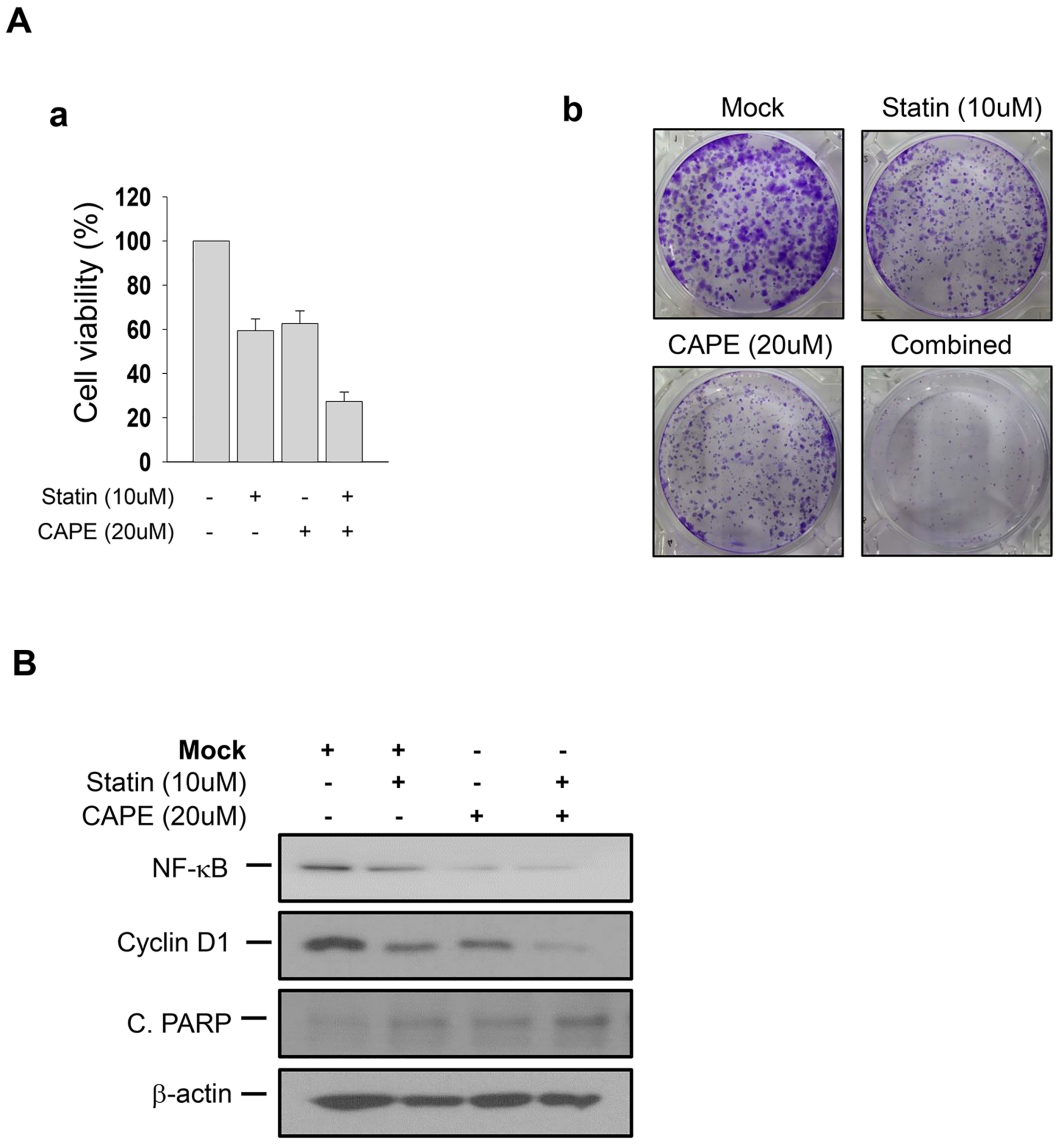
Synergistic anti-cancer effects of concurrent treatment with simvastatin and an NF-κB inhibitor in human CRPC cells. **A**. (a) Cell viability analysis according to treatment with simvastatin (10 μM) and *NF-κB* inhibitor CAPE (20 μM) in human CRPC cells (PC3). Values are presented as mean percentage of control ± S.E.M (*n* = 3). (b) Clonogenic assay according to the treatment with simvastatin (10 μM) and CAPE (20 μM) in PC3 cells. Crystal violet staining was conducted for visualizing colonies consisting of more than 50 cells. **B.** Protein expression patterns of the *NF-κB-Cyclin D1* signaling axis and the pro-apoptotic molecule cleaved-PARP according to the treatments of simvastatin simvastatin (10 μM) and CAPE (20 μM) in western blot analysis. The combination of treatments is presented as “+” or “-” and beta-actin was used as the loading control.

Conversely, the expression levels of the *let-7* miRNA family were dramatically upregulated by double treatment with simvastatin and CAPE (Fig 3B). We further confirmed that these synergistic effects were also induced by the genetic inhibition of *NF-κB* signaling using *p65*-siRNA transfection (Fig 3C). In this context, concurrent treatment with simvastatin and CAPE also synergistically suppressed cell viability and the number of colonies formed in these cells (Fig 4A). This synergistic effect was exerted by the downregulation of NF-κB and cyclin D1, and the subsequent induction of apoptotic cell death with upregulation of cleaved-PARP (Fig 4B). These data suggest that the *NF-κB* and *LIN28-let7-*miRNA signaling cascade acts as a key molecular mechanism underlying anti-cancer effects in human CRPC cells; therefore, concurrent treatment with simvastatin and the *NF-κB* inhibitor synergistically suppressed the growth of these cells.

## Discussion

In the present study, we observed that simvastatin suppressed *LIN28B*, which is a key player in tumorigenesis [9], and subsequently restored the *let7*-miRNA family as a novel mechanisms underlying the anti-cancer effects of simvastatin in human CRPC cells. *Lin28B*, a homologue of *LIN28*, was first identified as a key regulator of early development and stem cells [10]. Though the expression of *LIN28B* mostly disappears from adult tissues after the completion of development, various malignancies have shown significant *LIN28B* overexpression, thus suggesting its role as a potential oncogene [9,11,12]. Nadiminty and colleagues found that *LIN28* expression was enhanced in surgical specimens of human PCa, but its target tumor suppressor, *let-7c*, was downregulated [13]. Tummala *et al*. [14] also found that *LIN28* was highly expressed in human PCa specimens and PCa cell lines. They further demonstrated that *LIN28* knockdown suppressed tumor cell proliferation, whereas *LIN28B* overexpression promoted tumor cell growth, clonal proliferation, and invasiveness [14]. In agreement with previous reports, our data also showed that human CRPC cells, including both androgen sensitive and insensitive cells, showed significantly lower expression of the *let-7* miRNA family due to upregulation of the *LIN28B* gene.

Simvastatin has shown anti-cancer effects against various types of cancers in a number of preclinical and clinical studies [15–17]. For instance, Kamel *et al*. [18] found that simvastatin increased apoptotic cell death in human osteosarcoma cells by upregulating the AMPK and p38-MAPK pathways. Simvastatin also reduced cell viability and growth potential, along with significant attenuation of HMGCR expression and PSA secretion in human CRPC cells (C4-2) [19]. Furthermore, in both androgen receptor (AR)-sensitive (LNCaP and VCaP) and AR-insensitive CRPC cells (PC-3 and DU145), simvastatin was effective at inhibiting cell proliferation, clonogenic potential, and migration [20]. Consistently, our data also demonstrated that simvastatin treatment significantly reduced cell viability and colony formation in a dose-dependent manner in human CRPC cells (PC3).

Induction of apoptotic cell death by modulating the oncogenic signaling pathways, including JNK, Bax and Bcl-2, and PI3K/Akt and MAPK/ERK, has been suggested as the potential underlying mechanism of the anti-cancer effects of simvastatin [21–23]. Interestingly, our group has previously reported that simvastatin markedly inhibited the growth of human CRPC cells (PC3 and DU145) in a dose-dependent manner [7]. We suggested an increase in apoptotic cell death through the suppression of *NF-κB* activity as the crucial molecular mechanism underlying the effects of simvastatin on CRPC cells [7]. Likewise, Manu *et al*. also reported that simvastatin reduced the proliferation and invasion of human gastric cancer cells (SNU-5, SNU-16, MKN45, and AGS) through inhibition of the NF-κB signaling pathway [24]. Mechanistically, upregulation of the proto-oncogene tyrosine-protein kinase Src regulated by *NF-κB* triggered *LIN28B* transcriptional activation and subsequently reduced the expression of *let7*-miRNAs, as described by Iliopoulos and colleagues [25]. These transcriptional signatures were consistently observed in various human cancer cells as well as in cancer tissues [25].

Considering that simvastatin significantly repressed *NF-κB* activities in addition to the regulatory circuit of *NF-κB-LIN28-let7-miRNA*, the current study further identified that statins significantly inhibited the growth of human CRPC cells by suppression of *LIN28B* and subsequent upregulation of *let7*-*miRNAs*. More importantly, concomitant treatment with statins and the *NF-κB* inhibitor (CAPE) synergistically induced anti-cancer activities including the attenuation of proliferation and growth along with downregulation of *LIN28B* and *cyclin D1*, and restoration of *let7*-miRNA expression in human CRPC cells. To the best of our knowledge, this study offers novel molecular evidence for the anti-cancer effects of statins against human CRPC cells by regulating the *NF-κB-LIN28B-let7-miRNA* signaling pathway.

A number of limitations of our study should also be discussed. Firstly, we only used PC3 cells as the human CRPC model *in vitro*. Considering that different cell lines may show different phenotypic changes, various human CRPC cell lines, such as DU145 (androgen-insensitive), 22Rv.1, and C4-2B (androgen-sensitive), were further examined in a fashion similar to that used for PC3 in our study. Secondly, we only performed *in vitro* experiments to prove the mechanisms underlying the anti-cancer effects of statins in human CRPC. To provide more clear evidences for our findings, preclinical studies should be conducted using a human CRPC xenograft model. Third, our data focused on the effects of simvastatin and the concurrent inhibition of NF-κB as the master regulator of *LINB-let7-miRNA* axis. From the clinical perspective, the synergistic effects of simvastatin with novel AR targeting agents, such as enzalutamide and abiraterone acetate, in treating CRPC patients may receive more attention. A study by Gao *et al*. [26] recently showed that Lin28 induced secondary resistance to novel AR-targeting agents in PCa. Thus, our data can provide an insight into a way to overcome drug-resistance against novel agents targeting the AR pathway. Finally, to acquire more information for clinical application, we should screen the expression patterns of the *NF-κB-LIN28B-let7-miRNA* genes in specimens from patients with CRPC who were treated with statins, particularly to identify whether those patterns are correlated with oncological outcomes in such patients. From this, we can find a clue to extend our findings to clinical practice.

## Conclusions

In summary, human CRPC cells showed upregulated *LIN28B* expression, but significantly downregulated *let7-*miRNAs at the basal level. Accordingly, simvastatin treatment significantly reduced cell viability and clonal proliferation via induction of apoptosis mediated by suppressing *NF-κB* and *LIN28B* with consequent increasing *let7-miRNAs*. More importantly, concurrent treatment with simvastatin and an *NF-κB* inhibitor (CAPE) synergistically inhibited cell viability and the number of colonies formed. Therefore, our results indicate that the combination of simvastatin and CAPE can be a novel and alternative therapeutic approach for human CRPC treatment.

## Supporting information

S1 Fig Comparison of *LIN28B* expression levels in various human CRPC cells (PC3, 22Rv1 and C4-2B) compared to those in the normal prostate cell line (RWPE1) by (a) quantitative reverse transcriptase-polymerase chain reaction (qPCR) for *LIN28B* mRNA expression and (b) western blot analysis for protein expression. (TIF)

S2 Fig (a) Cell viability analysis according to different dosages of simvastatin (0, 5, 10, 20, and 40 μM) at 24 h in various human CRPC cells (PC3, 22Rv1 and C4-2B). (b) Clonogenic assay according to simvastatin treatment (20 μM for 24 h) in PC3, 22Rv1 and C4-2B cells.
(TIF)

S3 Fig Comparison of LIN28B expression levels by (a) western blot analysis and (b) the percentage of cell viabilities according to simvastatin treatment between the control and LIN28B-overexpression PC3 cells. (TIF)
