# Supplementary figures and images for: Concurrent treatment with simvastatin and NF-κB inhibitor in human castration-resistant prostate cancer cells exerts synergistic anti-cancer effects via control of the NF-κB/LIN28/let-7 miRNA signaling pathway
Source: PLoS One. 2017 Sep 14;12(9):e0184644. doi: 10.1371/journal.pone.0184644 (PMC5599006; doi:10.1371/journal.pone.0184644)

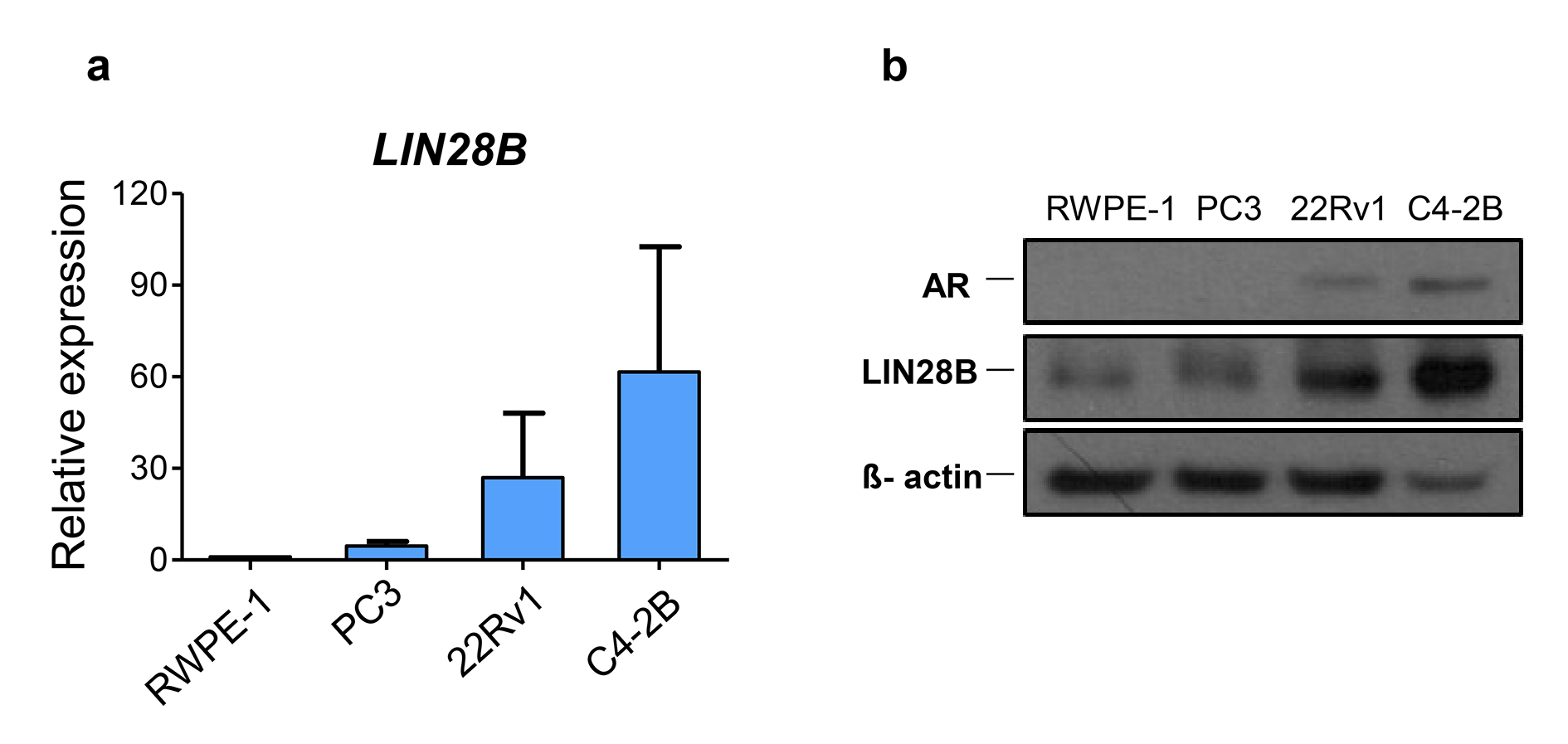

Supplement: S1 Fig — (TIF) [file pone.0184644.s001.tif]

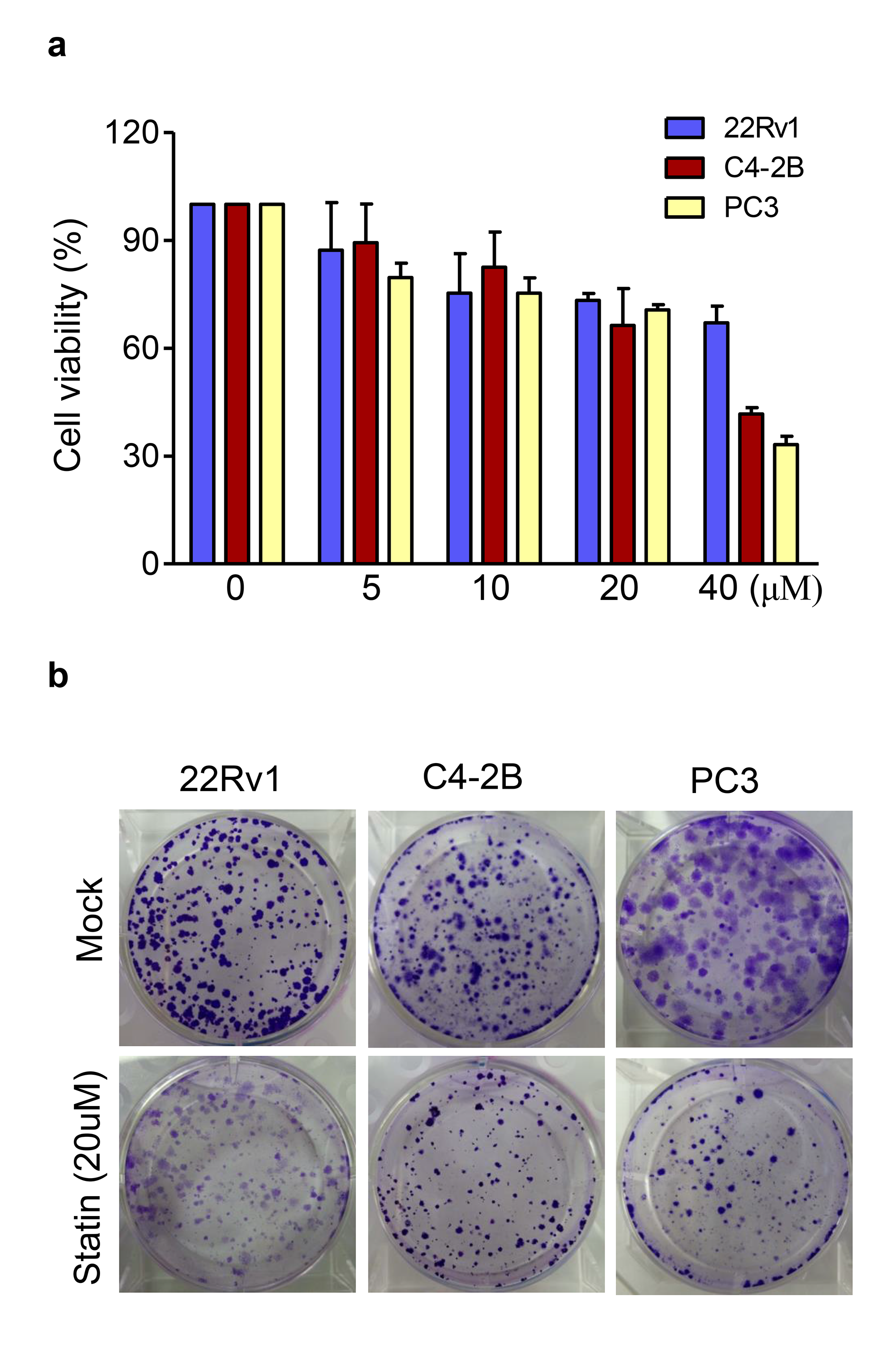

Supplement: S2 Fig — (a) Cell viability analysis according to different dosages of simvastatin (0, 5, 10, 20, and 40 μM) at 24 h in various human CRPC cells (PC3, 22Rv1 and C4-2B). (b) Clonogenic assay according to simvastatin treatment (20 μM for 24 h) in PC3, 22Rv1 and C4-2B cells. (TIF) [file pone.0184644.s002.tif]

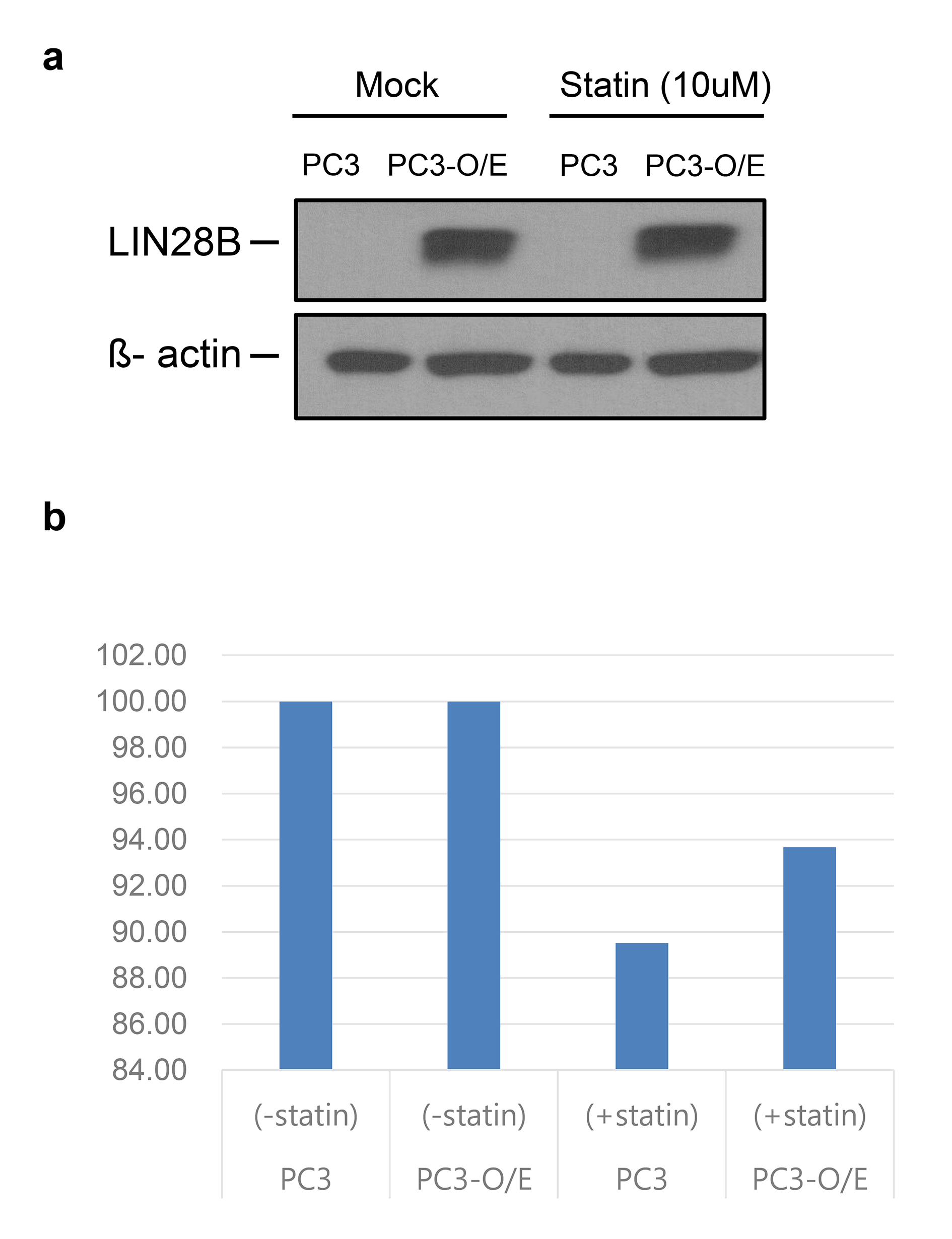

Supplement: S3 Fig — (TIF) [file pone.0184644.s003.tif]
